# Engineering Properties of Road Paving Mixtures with High Content of Reclaimed Asphalt and Recycled Waste Plastics

**DOI:** 10.3390/ma17235681

**Published:** 2024-11-21

**Authors:** Joseph Nicolas La Macchia, Orazio Baglieri, Davide Dalmazzo, Ezio Santagata

**Affiliations:** 1 Department of Environment, Land and Infrastructure Engineering, Politecnico di Torino, 10129 Turin, Italy; joseph.lamacchia@polito.it (J.N.L.M.); davide.dalmazzo@polito.it (D.D.); 2 Department of Civil and Environmental Engineering, Qatar University, Doha P.O. Box 2713, Qatar; ezio.santagata@qu.edu.qa

**Keywords:** reclaimed asphalt pavement (RAP), waste plastics, polymer-modified binder (PmB), asphalt mixtures, sustainability

## Abstract

Great efforts have been made in recent years by the scientific community and the asphalt industry in developing sustainable technologies for the production of asphalt mixtures for road paving applications, pursuing the use of ever higher quantities of recycled materials. In this regard, the challenge is to define the optimal formulation of the mixture which allows the various component materials to be synergistically combined without compromising the performance and durability of the asphalt pavement. In such a context, the experimental study described in this paper aimed to provide a contribution to research by investigating sustainable asphalt mixtures containing 50% reclaimed asphalt pavement (RAP) and polymeric compound composed of 100% recycled plastics. A wide set of mixtures was prepared in a laboratory by employing different dosages of polymeric compound added via the hybrid method at various binder contents. For comparison purposes, an additional set of reference asphalt mixtures containing standard polymer-modified binder (PmB) and virgin aggregate without RAP was prepared and tested. The experimentation focused on the main engineering properties of the asphalt mixtures, including their workability, volumetric properties, and mechanical characteristics. The experimental study involved a preliminary trial phase to establish an appropriate laboratory mixing procedure. The results obtained from the experimentation indicated that recycled waste plastics have good potential for use in asphalt mixtures with high contents of RAP, provided that the quantity of added plastics is adequately balanced.

## 1. Introduction

Great efforts have been made in recent years by the scientific community and the asphalt industry in the research and development of sustainable technologies for road paving applications, pursuing the deployment of ever higher quantities of recycled materials. In this context, reclaimed asphalt pavements (RAPs) and waste plastics have seen increasing use in the production of asphalt mixtures [1,2,3,4,5,6]. In this regard, the challenge is to define the optimal formulation of a mixture which allows the recycled materials to be synergistically combined with other components without compromising the overall performance and durability of the asphalt pavement.

Currently, it is common practice worldwide to incorporate 25–30% RAP in new hot-mix asphalt [7]. Despite the restrictions on technical specifications which may still be imposed by road agencies, the trend is to increase the RAP content even further [8,9]. High levels of RAP have a strong impact on the mechanical properties of the final product, mainly due to the aged binder [10,11]. In fact, the presence of a stiff and brittle binder results in stiffer and more brittle mixtures. This, on one hand, can improve the rutting resistance of asphalt pavements while, on the other hand, possibly having a negative impact on their fatigue performance and cracking susceptibility [3,4,12,13,14]. To overcome such problems, rejuvenating agents are commonly used to restore the original properties of aged binders. It has been demonstrated that the interaction mechanisms between the rejuvenator and aged bitumen entail a two-stage process, with an initial phase in which the rejuvenator agent coats the black aggregate and a subsequent phase characterized by gradual infiltration of the rejuvenator inside the binder thickness [15]. This process reduces the viscosity of the aged bitumen layer by layer and rebalances the existing chemical fractions of the binder. Through appropriate formulation of the asphalt mixture composition and an appropriate dosage of the rejuvenating agent, it is possible to produce hot-mix asphalt containing up to 50% RAP with fully satisfactory volumetric and mechanical properties [3]. The use of 50% RAP allows for saving approximately 2.5% virgin bitumen and 50% virgin aggregate, resulting in economic savings of approximately 40–45% related to the considered components [16].

Several studies have investigated the incorporation of waste plastics into asphalt mixtures, showing potential benefits in terms of stiffness, fatigue, rutting, and moisture damage performance [17,18,19]. In addition, the incorporation of plastics allows reducing the bitumen content, as the bitumen is replaced by the plastic itself [20,21]. However, the properties of the asphalt mixtures are significantly affected by the amount and type of plastic used [7].

Plastics can be incorporated into road pavements through either wet, dry, or hybrid methods, depending on the melting point of the material. In the wet method, low-melting plastics are added directly to bitumen and blended until a homogeneous binder phase is achieved [7,22,23]. In the dry method, high-melting plastics are added to the hot aggregate, partially replacing the stone particles prior to the addition of bitumen. The hybrid method replicates the process of the dry method but incorporates low-melting plastics which partially melt with the bitumen.

One of the main drawbacks of the wet method is the potential separation between the bitumen and polymer when the end product is stored and allowed to rest [20]. Conversely, the incorporation of plastics through dry and hybrid technologies can overcome compatibility and stability issues in the production of asphalt mixtures [24]. Several studies have shown significant improvements in the stiffness modulus and indirect tensile strength of asphalt mixtures containing plastics added through the dry method compared with those formed using the wet process [21,25]. It has been demonstrated that the incorporation of recycled plastics through hybrid and dry methods does not lead to additional environmental impacts compared with conventional technologies entailing the use of neat bitumen or PmB [26].

The synergistic use of a high RAP content and waste plastics has not been extensively investigated in the literature. Eskandarsefat et al. [27] compared the mechanical performances of dense-graded base/binder course mixtures modified with a recycled plastic containing graphene nano-platelets added by the dry process and a reference mix of SBS PmB. In this case, only 10% RAP was employed. The mixture modified via the dry process showed increased tenacity, stiffness, and resistance to permanent deformation, demonstrating the effectiveness of this technology as a viable alternative to the conventional wet process. Saadeh et al. [28] characterized the properties of asphalt mixtures modified with recycled, linear low-density polyethylene and reactive elastomeric terpolymers, using aggregate with 20% RAP. They found a reduction in fatigue cracking resistance for the mixtures containing a polymer-modified binder and RAP aggregate, while similar resistance to rutting was observed regardless of the aggregate type and asphalt modification. Hayat et al. [29] used up to 40% RAP together with shredded plastic waste derived from PET added through the wet method. The outcomes demonstrated an enhancement in the rutting resistance of the asphalt mixtures. Di Mino et al. [30] investigated the multi-recyclability of mixtures containing 50% RAP both with and without polymeric compounds added via the dry method. They concluded that the incorporation of recycled plastics does not compromise the recyclability of asphalt mixtures, even after several recycling cycles.

As a part of a wider research project, the experimental study described in this paper aimed to investigate sustainable asphalt mixtures containing 50% RAP and a polymeric compound sourced from waste plastics. The addition of the polymeric compound was carried out using the hybrid method at different dosages. The production of asphalt mixtures involved the development and fine-tuning of a specific laboratory mixing procedure. The testing program entailed determining the engineering properties of the mixtures, including the workability, volumetrics, and mechanical characteristics. For comparison purposes, additional control asphalt mixtures containing standard PmB and virgin aggregate without RAPs were prepared and tested. An analysis of the results was carried out to highlight the combined effects of RAPs and plastics on the mixture performance as a function of the variables under consideration.

## 2. Asphalt Mixtures and Experiment Design

### 2.1. Base Materials

The asphalt mixtures investigated in this study were produced by combining different base materials which included virgin aggregates, single-source RAP, a polymer compound derived from waste plastics, two asphalt binders, and a bio-based rejuvenating agent.

The virgin aggregate was of siliceous origin and was supplied by a local quarry (Bitux S.p.A., Foglizzo, Italy) in three different sizes (0/5, 8/16, and 16/20). It was subjected to a preliminary characterization consisting of sieve analysis and determination of the apparent specific gravity according to EN 933-1 [31] and EN 1097-6 [32], respectively.

The RAP was collected from the same supplier as the virgin aggregate. The material came from the demolition and milling of aged pavements on a major motorway located in northwest Italy. The RAP was screened into two fractions (RAP 0/12 and RAP 0/20) and stored in cone-shaped stockpiles, placed on a paved surface, and covered to prevent contamination as well as minimize water infiltration. The binder contents of both RAP fractions were determined by means of the ignition technique according to EN 12697-39 [33]. The results indicated binder contents of 4.15 and 5.45% by weight of the aggregate for RAP 0/12 and RAP 0/20, respectively. The aggregate recovered from the ignition process was subsequently tested to determine its particle size distribution and apparent specific gravity, following the same standard procedures mentioned above.

**Figure 1:**
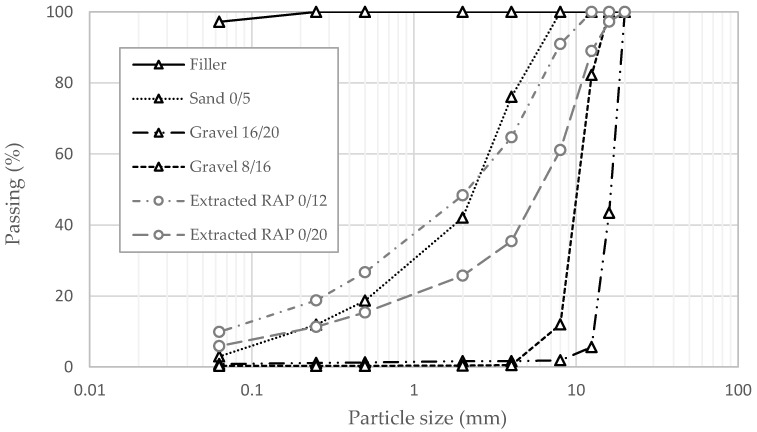
Particle size distributions of aggregate and RAP fractions.

The gradation curves and the values of the apparent specific gravity obtained for the virgin aggregate and the RAP fractions are shown in Figure 1 and Table 1, respectively.

The polymeric compound was composed of recycled hard plastics specifically formulated for incorporation within asphalt mixtures via dry and hybrid methods. The recycled plastics came from urban and industrial waste subjected to an undiscovered processing scheme entailing washing, separation, and grinding, which led to a final material consisting of 4–6 mm plastic granules. The polymeric compound used in this study was supplied by Iterchimica S.p.A. (Suisio, Italy) and is protected by an industrial patent, making its exact formulation proprietary and undisclosed. The main properties of such a material are listed in Table 2.

The asphalt binders, collected from the same supplier as the aggregate, were 50/70 penetration grade bitumen (Binder A), classified according to EN 12591 [34], and SBS PmB 45/80-70 modified binder (Binder B), classified according to EN 14023 [35]. Binder A was used as the base bitumen for mixtures modified with recycled plastics, while Binder B was chosen as a reference standard modified bitumen for comparison purposes. The performance grades (PGs) of the two binders, determined according to the Superpave classification system (AASHTO M323-22) [36], are shown in Table 3, together with the empirical characteristics expressed in terms of penetration (EN 1426) [37] and softening point (EN 1427) [38].

The rejuvenator used in this study was a bio-based agent, whose properties are given in Table 4. It was added in a single dosage equal to 0.3% by the weight of the RAP, as recommended by the manufacturer which provided the material (Iterchimica S.p.A., Suisio, Italy).

### 2.2. Composition of Mixtures

**Figure 2:**
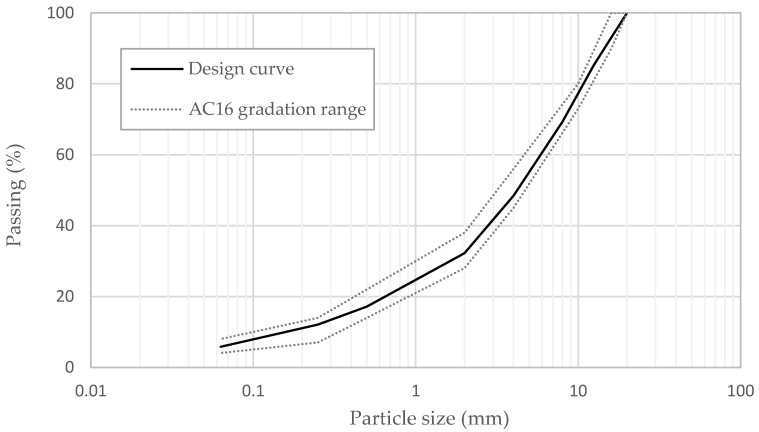
Gradiation limits and target gradation curve of AC mixtures.

The type of asphalt mixture selected for the experimental study was characterized by a maximum nominal aggregate size of 16 mm (AC16), with a particle size distribution determined according to Italian technical specifications for standard dense-graded binder courses [39]. The target gradation curve and the gradation limits adopted are displayed in Figure 2. The total amount of RAP was added at a rate of 30 and 20% by weight of the aggregate for RAP 0/12 and RAP 0/20, respectively.

Two sets of asphalt mixtures modified with waste plastics were prepared in a laboratory using two different contents of polymeric compound equal to 0.3 and 0.5% by weight of the bulk mixture. The two sets of mixtures were referred to as ACR-SP1 (0.3%) and ACR-SP2 (0.5%). A set of control mixtures containing PmB (ACR-PMB) was also investigated in order to discriminate the effects of polymeric modification via the hybrid method compared to those of standard modification via the wet method. Different binder dosages were used for each set of mixtures to evaluate the effect of the binder on the workability and mechanical properties. It is worth noting that the rejuvenator and the polymeric compound were computed in the total binder content in accordance with the hypothesis of considering them as binder extenders which actively contributed to the “binding” of the lithic structure.

An additional control mixture (AC-SP1) containing 0.3% polymeric compound and virgin aggregate only was finally considered to highlight the influence of the RAP.

According to the experimental design described above, a total of 13 different asphalt mixtures were manufactured and tested. Their descriptions and compositions are summarized in Table 5.

### 2.3. Mixing Procedure

A major issue in the production of asphalt mixtures containing RAP is how to incorporate the reclaimed asphalt and the rejuvenating agent in the asphalt mixing plant. In fact, inadequate mixing times and temperatures adopted in the production process may lead to insufficient interaction between the aged and virgin bitumen, thus resulting in poor properties for the final mixture [4]. Three main methods of RAP addition are commonly used, depending on the type and technological features of the plant:

- Cold RAP addition in the mixer, used in batch mixing plants;
- Cold RAP addition into the dryer, used in both batch and continuous mixing plants;
- Preheated RAP addition, used in batch mixing plants equipped with a separate drum.

Different methods allow for different RAP inclusion limits and require different virgin aggregate heating temperatures to account for the percentages and temperatures of the RAP added to the mixture.

Rejuvenator can either be pre-mixed with the virgin binder or sprayed directly onto the RAP [8]. In the case of asphalt batch plant, Zaumanis et al. [40] suggested that direct contact between the RAP bitumen and the rejuvenator is optimal, provided that sufficient contact time is allowed to promote activation of the RAP binder. In the case of laboratory mixing, in the study conducted by Rathore and Zaumanis [41] on mixtures containing high levels of RAPs, no significant differences in properties were observed regarding whether the rejuvenating agent was pre-blended with virgin bitumen or sprayed directly onto the reclaimed asphalt. A variety of procedures can be found in the literature to prepare mixtures containing RAPs in a laboratory [42,43,44]. Several studies discouraged heating RAPs in an oven for more than 2 h to prevent further aging of the material [42,45]. Cheng et al. [46] provided specific heat capacity data for raw materials used in asphalt mixtures to determine the appropriate heating temperature for RAPs.

Various procedures have also been proposed for incorporating plastics into asphalt mixtures. Russo et al. [24] blended a polymeric compound with virgin aggregate at 180 °C and kept both materials in an oven for 45 min to facilitate the softening of the granules before adding the hot bitumen. In contrast, Boom et al. [47] mixed plastics at room temperature with virgin aggregate heated to 180 °C for 5 min to allow the plastics to melt. Vasudevan et al. [20] suggested spraying plastics onto heated aggregate to promote melting and subsequent coating of the stone particles.

**Figure 3:**
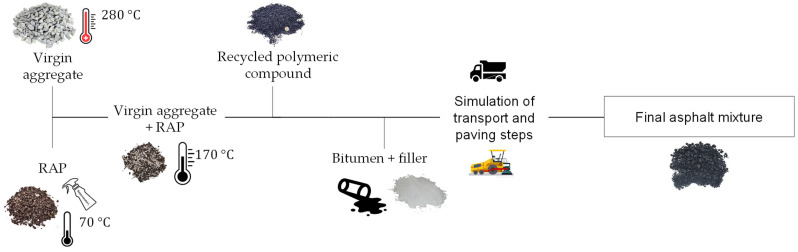
Schematic flowchart of the mixing procedure developed.

The laboratory mixing procedure adopted in this study was defined on the basis of preliminary trials in order to optimize the simultaneous use of RAPs and plastics. The procedure consisted of several sequential steps, as outlined in the following and shown in Figure 3:

- The RAP was preheated to 70 °C for a maximum of 2 h. It was spread on sufficiently large trays to maximize exposure to the rejuvenating agent, which was sprayed using a spray bottle directly onto the RAP during the heating phase.
- The virgin aggregate was heated to 280 °C and then blended with the RAP. The thermal shock caused by the contact between aggregate and RAP allowed both to reach the desired mixing temperature, set equal to 170 °C. The increase in temperature made the RAP softer, breaking up aggregate clusters and improving blending with the aggregates. The contact time was limited to 1 min to ensure effective temperature transfer without excessive exposure, which could alter the material properties and cause drain down of the RAP binder.
- The polymeric compound was then introduced and mixed with the virgin aggregate and RAP for a further 2 min before the addition of filler and bitumen.
- When mixing was complete, the asphalt mixture was held in the mixer for 15 min to simulate the transport phase at the paving site.
- Finally, the blend was further mixed for 1.5 min to simulate the action of a paver.

## 3. Testing and Results

The experimental investigation conducted on asphalt mixtures included the evaluation of their workability properties and performance-related characteristics, both volumetric and mechanical.

A description of the laboratory methods and a discussion of the results are presented in the following sections.

### 3.1. Workability

The term “workability” is generally used to describe the ease with which asphalt mixtures can be laid, handled, and compacted. In this study, the workability of asphalt mixtures refers to the ease with which the density of the mix can be increased during compaction operations by external pressure.

The workability properties of the investigated mixtures were assessed by means of the gyratory shear compaction technique. The adopted method entailed the compaction of specimens 150 mm in diameter according to EN 12697-31 [48], with an applied normal pressure of 600 kPa and an axial inclination of 1.25°.

The advantages of gyratory shear compaction are related not only to its ability to simulate the actual compaction mechanism produced by rollers in the field but also the possibility of monitoring the densification process on a quantitative basis. The parameters used to evaluate and compare the workability properties were obtained by relating the percentage of compaction (C) to the number of gyrations (N) according to the following expression:

C = C1 + k log N(1)

where C1 is the self-compaction and k is the compaction rate. The C1 parameter provides a measure of the initial settlement of the material after laydown; mixtures which settle too much are considered to be tender during the construction phase and unstable when subjected to traffic. The parameter k relates to the rate of the compaction process and the number of passes needed to reach the target density. The higher the value of k, the faster the compaction process.

An additional parameter considered in this study was the compaction densification index (CDI), introduced by Bahia et al. [49]. The CDI is calculated as the area under the compaction curve from the eighth rotation up to the rotation corresponding to 92% compaction. The index indicates the effort required by the rollers during the compaction phase to reach 8% voids in the field (open to traffic condition). The lower the CDI, the better the workability of the mixtures during pavement construction operations.

**Figure 4:**
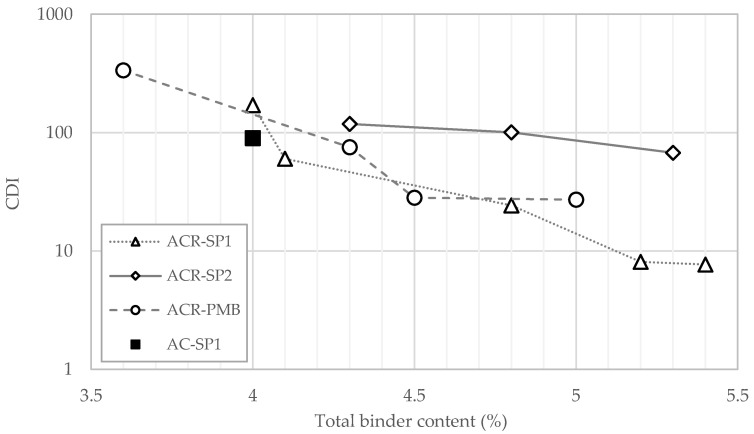
CDI values of the asphalt mixtures.

The results obtained from the analysis of the gyratory data points are shown in Table 6 (C1 and k) and Figure 4 (CDI).

A slight increase in C1 values with an increasing binder content was observed for all of the mixtures considered. This was in line with our expectations, since a higher amount of binder made the mixture more “fluid”, thus promoting its aptitude for self-compacting.

In the case of the k parameter, the data reveal that the rate of compaction was not significantly sensitive to variation in the binder dosage. This is also compliant with our expectations since the slope of the compaction curve is mostly governed by the lithic structure, which was the same for each set of mixtures. By comparing the values of C1 and k for the different materials, it can be noticed that the differences between one set of mixtures and the others were quite small.

Conversely, the differences appeared to be more relevant when the CDI parameter was considered, indicating that this index was more effective in evaluating the workability properties of the mixtures. In particular, a clear distinction can be made between the CDI curve corresponding to the ACR-SP2 group with respect to those of the ACR-SP1 and ACR-PMB groups. The higher compaction effort required for the ACR-SP2 mixtures was evidently related to the higher viscosity of the binder phase due to the higher dosage of plastics. Moreover, the CDI values were less sensitive to the binder content for these mixtures compared with the others. As a result, in the range considered in this study, the gap between ACR-SP2 and the other mixtures increased at increased percentages of the total binder.

Similar trends were observed when comparing the ACR-SP1 and ACR-PMB curves, revealing that the effects of the standard SBS polymer incorporated via the wet method and those of 0.3% plastics incorporated via the hybrid method were substantially equivalent.

### 3.2. Volumetric Characteristics

The volumetric characterization of the asphalt mixtures was carried out on gyratory cylindrical specimens and consisted of the determination of their air voids (v), voids in the mineral aggregate (VMA), and voids filled with bitumen (VFB) according to EN 12697-8 [50]. The determination of the volumetric parameters of the compacted samples required the determination of the theoretical maximum density (TMD) of the loose mixtures, carried out by means of the pycnometer method according to EN 12697-5 [51].

**Figure 5:**
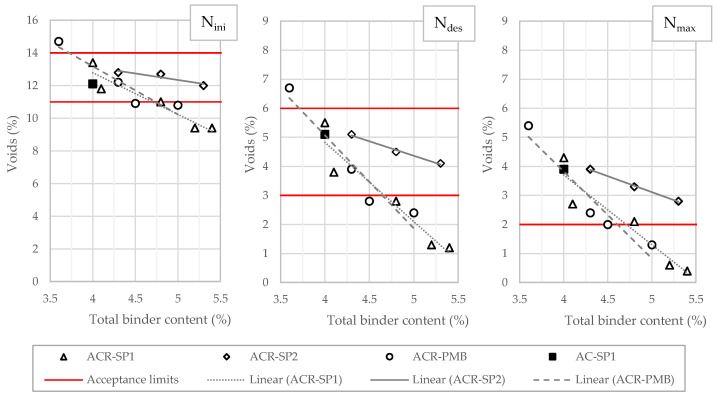
Air void content as a function of the binder dosage of the mixtures at different numbers of gyrations.

The v values were obtained at different numbers of gyratory rotations, set equal to 10 (N_ini_), 120 (N_des_), and 200 (N_max_). N_ini_ is the number of gyrations intended to reproduce the initial density of the mix, N_des_ is the number of gyrations required to produce a density equivalent to the expected in-place density after compaction, and N_max_ is the number of gyrations considered to ensure that the mixture does not densify too much under traffic to avoid potential rutting phenomena. The graphs in Figure 5 illustrate the variation in voids as a function of the binder contents of the different mixtures. The graphs also show the specification limits defined by Italian technical standards, which were taken as a reference. The v values decreased with an increasing total binder content, with the curves corresponding to the ACR-PMB and ACR-SP1 blends showing quite similar trends at all gyration levels. This indicates that the combination of neat Binder A and 0.3% waste plastics produced equivalent effects in terms of the sensitivity of the volumetric properties to variation in the binder content compared with the standard modified Binder B. It is worth noting that data points corresponding to the AC-SP1 mixture containing 0.3% waste plastics with no RAPs perfectly fit both curves, indicating that the presence of reclaimed asphalt had no influence on the degree of compaction. The use of 0.5% plastics (ACR-SP2) resulted in a significantly different response. The reduced slope of the curves indicates a reduced dependence on the binder dosage. In addition, the curves appear to be vertically shifted upward compared with the others (ACR-PMB and ACR-SP1). This implies that more virgin bitumen is required to achieve predefined degrees of compaction and to compensate for the stiffening effect consequent from the higher dosage of plastics added to the blends.

The VMA and VFB values were determined at N_des_. The VMA parameter represents the intergranular void space between aggregate particles in the compacted asphalt mixture, including the volume of the air voids and the volume of the effective binder covering the particles. The purpose of controlling the VMA is to ensure enough space to accommodate an adequate thickness of the bituminous film, thus providing adequate cohesion and durability to the mixture. The VFB parameter represents the percentage of VMA filled with effective bitumen and gives a measure of the actual thickness of the bituminous film covering the aggregate particles. Low VFB values indicate mixtures which may lack durability, while high VFB values may result in an unstable material with a potential tendency to bleed.

The results obtained for the VMA and VFB, together with the TMD values, are summarized in Table 7.

A minimum limit of 13% for the VMA was considered in the technical specification assumed as a reference, while a control interval between 69 and 73% was considered for the VFB. The total binder dosage needed for the various types of mixtures to meet the reference volumetric requirements varied within different intervals. By matching the limits indicated above and considering that part of the total binder was provided by the RAP, the actual virgin bitumen needed spanned between 1.4 and 2.2% for ACR-PMB, 1.6 and 2% for ACR-SP1, 2 and 3% for ACR-SP2. This indicates that the incorporation of 0.3% plastics contributed to the largest saving in the fresh binder. This outcome is fully coherent with those which emerged from the workability analysis.

### 3.3. Mechanical Characteristics

The mechanical characterization of the asphalt mixtures consisted of the evaluation of their stiffness and strength. The stiffness properties were determined by means of indirect tensile modulus (ITM) tests carried out according to EN 12697-26 Annex C [52] at 10, 20, and 40 °C. The strength properties were determined by means of indirect tensile strength (ITS) tests performed according to EN 12697-23 [53] at 25 °C. All measurements were run in two replicates. The coefficient of variation (CoV) obtained in the majority of cases was less than 5%, which was considered acceptable for the purpose of this study.

**Figure 6:**
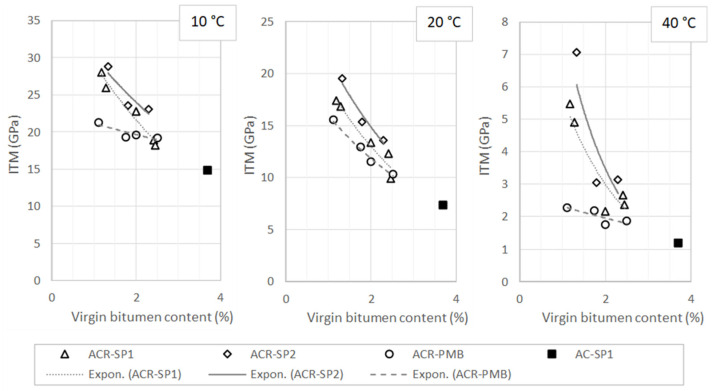
Stiffness moduli at different temperatures for the asphalt mixtures under investigation.

Figure 6 shows the ITM values plotted against the virgin bitumen content of the mixtures tested at various temperatures.

It was observed that the stiffness moduli decreased with increasing binder dosages for all materials at all temperatures. It was also observed that the ACR-SP2 mixtures exhibited the highest stiffness, followed by ACR-SP1 and ACR-PMB. The differences were more pronounced at a temperature of 40 °C, especially when comparing the ACR-SP1 and ACR-PMB groups. This result can be attributed to the higher amount of polymer present in the hybrid blends compared with the wet blends. The diagrams also report the ITM parameters obtained for the AC-SP1 mixture, indicating lower values with respect to other ones due to the absence of an RAP. However, it is interesting to note that curves of both the ACR-SP1 and ACR-PMB mixtures tend to reach the points corresponding to AC-SP1 as the content of virgin bitumen increased. The increase in virgin binder is associated with a progressive replacement of the RAP with virgin aggregate, which ultimately led to the mixtures having the same characteristics.

Enhanced stiffness properties of mixtures contribute to the structural response of the pavement under traffic loads, in terms of bearing capacity and stress–strain behavior. However, it has been pointed out that excessive stiffness can make mixtures too brittle and prone to premature fatigue failure. Controlling stiffness is thus essential to ensure adequate pavement performance in the field. In the specific case of the mixture types investigated in this study, another crucial aspect was to distinguish the stiffening effects caused by the polymeric compound from those caused by the RAP.

**Figure 7:**
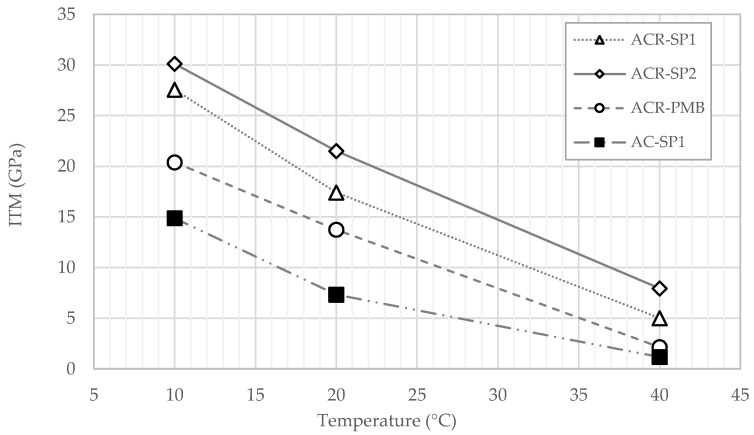
Comparison of ITM values of mixtures with 4% total binder content at different temperatures.

In this regard, a regression analysis was carried out to interpolate data points and to describe the response of the mixtures as a function of the binder content. The power law equations obtained from the analysis (omitted for the sake of conciseness) were then used to predict the stiffness modulus of the materials at a fixed total binder content, thus eliminating possible biasing effects related to mix composition factors. The selected value of the binder content was set equal to 4%, and the corresponding stiffness moduli are reported in Figure 7.

The results confirm that ACR-SP2 was the most rigid mixture at all temperatures, followed in order by ACR-SP1, ACR-PMB, and AC-SP1. Moreover, the curves are almost parallel, indicating a relative difference in stiffness moduli that was quite constant with the temperature.

Pairwise comparison of the mixtures allowed discriminating the effects of different factors considered in the investigation. The lower ITM values of ACR-PMB compared with those of ACR-SP1 and ACR-SP2 indicate a less pronounced stiffening effect of SBS polymer modification with respect to modification with plastics, which is ascribable to the softer bituminous phase of PmB. The difference between ACR-SP2 and ACR-SP1 indicates that the increase from a 0.3% to 0.5% plastic dosage resulted in a relative increase in stiffness of about 22–23%. The difference between ACR-SP1 and AC-SP1, which had the same composition except for the use of solely virgin aggregate in the second one, can evidently be attributed to the RAP.

Based on the results presented above, it can be argued that the use of reclaimed asphalt and waste plastics needs to be properly balanced in order to achieve a satisfactory level of stiffness. For the materials investigated in this study, the combination of 50% RAP and 0.3% waste plastic resulted in a mixture which was not excessively stiff and well within the standard interval of 8000–15,000 MPa suggested by the Italian specifications adopted as a reference.

**Figure 8:**
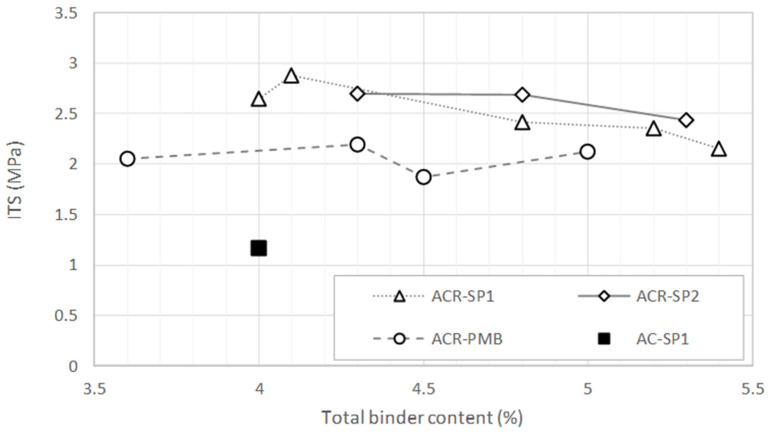
ITS values of the asphalt mixtures.

Figure 8 displays the results obtained from the ITS tests. Although the data presented in the diagram do not show a clear trend in the tensile strength as a function of the binder dosage, it is possible to discriminate between the different types of mixtures based on the average ITS levels. Mixture AC-SP1 produced with virgin aggregate exhibited lower strength values compared with the other mixtures, confirming the prominent role of reclaimed asphalt in the mechanical properties of the material. In the case of the mixtures containing RAPs, the blends produced using the hybrid method showed greater strength than those produced using the wet method. Moreover, increasing the polymeric compound content from 0.3 to 0.5% resulted in no significant increase in the ITS values.

## 4. Summary and Concluding Remarks

The experimental study described in this paper explored the synergistic use of reclaimed asphalt and recycled waste plastics for the production of sustainable asphalt mixtures. The stiffening effects produced by the combination of RAPs and the polymeric compound posed a challenge in the formulation of mixtures to make them sufficiently workable and characterized by adequate performance-related characteristics. For this reason, sustainable asphalt mixtures containing 50% RAP and a polymeric compound from recycled waste plastics added via the hybrid method were compared with control asphalt mixtures containing standard PmB. Furthermore, another asphalt mixture containing the polymeric compound and only virgin aggregate was manufactured to discriminate the effect of RAPs. The laboratory investigation focused on evaluating the key engineering properties of the mixtures, including the compaction characteristics, volumetrics, stiffness, and strength. A laboratory mixing procedure was developed to prepare the asphalt mixtures investigated.

Notwithstanding the predominant role of RAPs in the mechanical properties of the tested mixtures, the incorporation of recycled waste plastics via the hybrid method led to further increases in stiffness and strength compared with the mixture modified with SBS polymer.

The results obtained from the experimentation indicated that the addition of 0.3% recycled plastics resulted in mixtures having adequate workability and volumetric properties, with stiffness values compliant with the assumed reference limits.

Conversely, the use of 0.5% plastics reduced the workability properties of the materials and led to relatively high stiffnesses, with a possible negative impact on pavement field performance.

It can be concluded that recycled waste plastics have good potential for use in asphalt mixtures with high RAP contents, provided that the quantity of added plastics is adequately balanced.

Further research is needed to investigate a wider array of materials, including reclaimed asphalts from different sources and different rejuvenating agents. The performance characteristics of mixtures in terms of fatigue and rutting resistance should also be considered in future work.

## Figures and Tables

**Table 1:** Apparent specific gravity of aggregate and RAP fractions.

	Apparent Specific Gravity ρ_mv_ (Mg/m^3^)
Sand 0/5	2.780
Gravel 8/16	2.849
Gravel 16/20	2.813
Extracted RAP 0/12	2.765
RAP 0/12	2.538
Extracted RAP 0/20	2.774
RAP 0/20	2.587

**Table 2:** Main characteristics of the polymeric compound additive used in this study.

Aspect	Granules
Color	Shades of gray
Apparent density at 80 °F (25 °C)	0.4–0.6 g/cm^3^
Softening point	160–180 °C

**Table 3:** Characteristics of asphalt binders.

	Performance Grade	Penetration at 25 °C (dmm)	Softening Point (°C)
Binder A	PG 46E-22	70	48.1
Binder B	PG 64E-22	55	80.3

**Table 4:** Properties of the rejuvenating agent.

Aspect	Liquid
Color	Brown–purple
Density at 80 °F (25 °C)	0.93 ± 0.1 g/cm^3^
Viscosity at 80 °F (25 °C)	100 ± 50 cP
Flash point	>390 °F (200 °C)
Water content	<2%

**Table 5:** Characteristics of the manufactured asphalt mixtures.

Mixture Code	RAP (%) (by Weight of Aggregate)	Binder Type	Total Binder (%)	Virgin Bitumen (%)	RAP Bitumen (%)	Rejuvenator (%)	Polymeric Compound Content (%)
ACR-SP1 4.0	50	A	4.00	1.18	2.37	0.15	0.30
ACR-SP1 4.1	50	A	4.10	1.29	2.36	0.15	0.30
ACR-SP1 4.8	50	A	4.80	2.00	2.35	0.15	0.30
ACR-SP1 5.2	50	A	5.19	2.42	2.32	0.15	0.30
ACR-SP1 5.4	50	A	5.42	2.46	2.51	0.15	0.30
ACR-SP2 4.3	50	A	4.34	1.33	2.36	0.15	0.50
ACR-SP2 4.8	50	A	4.80	1.80	2.35	0.15	0.50
ACR-SP2 5.3	50	A	5.29	2.30	2.34	0.15	0.50
ACR-PMB 3.6	50	B	3.64	1.11	2.38	0.15	0.00
ACR-PMB 4.3	50	B	4.26	1.75	2.36	0.15	0.00
ACR-PMB 4.5	50	B	4.50	2.00	2.35	0.15	0.00
ACR-PMB 5.0	50	B	5.00	2.51	2.34	0.15	0.00
AC-SP1 4.0	0	A	4.00	3.70	0.00	0.00	0.30

**Table 6:** Parameters of the compaction process.

Mixture Code	C1	k
ACR-SP1 4.0	80.0	7.0
ACR-SP1 4.1	81.6	7.0
ACR-SP1 4.8	82.6	7.1
ACR-SP1 5.2	84.3	6.9
ACR-SP1 5.4	84.3	7.0
ACR-SP2 4.3	80.7	6.8
ACR-SP2 4.8	80.5	7.2
ACR-SP2 5.3	81.5	6.9
ACR-PMB 3.6	77.9	7.4
ACR-PMB 4.3	80.8	7.3
ACR-PMB 4.5	82.2	7.2
ACR-PMB 5.0	82.4	7.3
AC-SP1 4.0	81.9	6.2

**Table 7:** TMD, VMA, and VFB values of the asphalt mixtures.

Mixture Code	TMD (Mg/m^3^)	VMA (%)	VFB (%)
ACR-SP1 4.0	2.598	15.2	63.9
ACR-SP1 4.1	2.573	13.8	72.5
ACR-SP1 4.8	2.558	14.6	81.0
ACR-SP1 5.2	2.542	14.1	91.1
ACR-SP1 5.4	2.532	14.6	91.8
ACR-SP2 4.3	2.567	15.6	67.2
ACR-SP2 4.8	2.554	16.1	72.2
ACR-SP2 5.3	2.542	16.9	75.7
ACR-PMB 3.6	2.615	15.5	56.9
ACR-PMB 4.3	2.594	14.4	73
ACR-PMB 4.5	2.576	14.0	79.9
ACR-PMB 5.0	2.555	14.8	83.6
AC-SP1 4.0	2.585	14.8	65.6

## Data Availability

The data presented in this study are available on request from the corresponding author (the data are not publicly available due to privacy concerns).
